# The impact of pine wilt disease on the endophytic microbial communities structure of *Pinus koraiensis*

**DOI:** 10.3389/fmicb.2024.1493808

**Published:** 2024-10-25

**Authors:** Debin Li, Yuezhen Yu, Chuan Tian, Shisong Lu, Shengwei Jiang

**Affiliations:** ^1^College of Life Engineering, Shenyang Institute of Technology, Fushun, China; ^2^Key Laboratory of Nation Forestry and Grassland Administration on Northeast Area Forest and Grass Dangerous Pest Management and Control, Fushun, China; ^3^Liaoning Provincial Key Laboratory of Dangerous Forest Pest Management and Control, Fushun, China; ^4^College of Forestry, Shenyang Agricultural University, Shenyang, China; ^5^Liaoning Forestry and Grassland Bureau, Shenyang, China

**Keywords:** pine wood nematode, endophytes, abundance, microbial diversity, pines

## Abstract

Pine Wilt Disease (PWD) is a devastating pine tree disease characterized by rapid onset, high mortality rate, quick spread, and difficulty in control. Plant microbiome plays a significant role in the development of PWD. However, the endophytic microbial communities of *Pinus koraiensis* infected by pine wood nematode (PWN) *Bursaphelenchus xylophilus* remain largely unexplored. In this study, we analyzed the structural changes of endophytic communities of *P. koraiensis* after infection by the PWN using high-throughput sequencing technology. The results showed that the community structure underwent significant changes as the degree of PWN infection intensified. The diversity and abundance of endophytic fungi in *P. koraiensis* increased, while those of endophytic bacteria in *P. koraiensis* decreased during the infection process. Meanwhile, the abundance of some dominant microorganisms has also changed, including species such as *Graphilbum* and *Pseudoalteromonas*. Functional prediction analysis showed that the functional composition of endophytic fungi in *P. koraiensis* was significantly different across the development of PWD, while the composition of endophytic bacteria remained essentially similar. The results indicated that PWN infection had a significant impact on the structure, diversity, abundance, and functional gene composition of endophytic microbial communities in *P. koraiensis*, and most of the main endophytic microbial groups tended to coordinate with each other. This work provides a better understanding of the changes in endophytic community structure and function caused by PWD infection of *P. koraiensis*, which may benefit the exploration of potential endophytes for PWN biocontrol.

## 1 Introduction

Pine wood nematode disease (PWD) is a global disease that poses a significant threat to the health of Pinaceae plants (Feng et al., 2022; Zhang and Zhang, 2024). The etiology of PWD is still not well understood at present, and the prevention and control of this disease have long been a challenging problem worldwide (Wang, 2004; Ye and Wu, 2022; Li, 2022; Cardoso et al., 2024). The pathogen of PWD, pine wood nematode (PWN) *Bursaphelenchus xylophilus* (Steiner and Bubrer) Nickle, belongs to the family Aphelenchoididae and the genus *Bursaphelenchus* (Yan and Tan, 2019). In China, the PWN is listed as the only national first-class forest pest. Pines are important trees with both ecological and economic value in China, mainly including *Pinus massoniana*, *P. tabuliformis*, *P. koraiensis*, and *P. thunbergia* (Pan, 2023). In particular, *Pinus koraiensis* (Siebold and Zucc) wood has been widely used in various construction projects and is considered to be a high-quality timber for construction in China (Wang et al., 2024). Aside from wood products, pine trees can provide a variety of non-wood products, such as resin, biofuels, and pine nuts (Neis et al., 2019; Kaviriri et al., 2021). However, most of these pine trees are susceptible to PWD (Pan, 2023). According to the official data from the National Forestry and Grassland Administration in 2023, PWD has spread to more than 700 counties across 19 provinces in China, with an affected area reaching 1.22 × 10^6^ hm^2^. About 7.6 million pine trees died this year due to PWD (Liu B. et al., 2024; Liu M. J. et al., 2024).

PWD entered the northeastern region of China in 2016, where major economic tree species, such as *Pinus koraiensis*, were subsequently identified as host plants, leading to significant damage. In particular, *P. koraiensis*, with its high susceptibility and rapid onset of disease, has suffered particularly severe harm (Fang, 2021; Lu, 2024). Plant endophytes are an important component of plant life, playing a very significant role. They are involved in various important physiological activities of plants, promoting growth and development, enhancing plant’s tolerance to external environmental stimuli, strengthening plant stress resistance, and are beneficial for biological pest control, as well as the bioremediation of contaminated soils (Wen et al., 2022; Han et al., 2024; Yang et al., 2024; Liu B. et al., 2024; Liu M. J. et al., 2024).

As a main host of the widely distributed PWN in China, however, the microbial communities associated with *P. koraiensis* remain largely unexplored. To the best of our knowledge, research on the overall endophytic communities of *P. koraiensis* has not been reported. In this study, we utilized high-throughput sequencing to investigate the communities of endophytes in healthy *P. koraiensis* (PKh), as well as those *P. koraiensis* naturally infected by *B. xylophilus* at the early stage (PKe) and *P. koraiensis* naturally infected by *B. xylophilus* at the last stage (PKl) under natural conditions. The dynamic changes in the structure of bacterial and fungal communities within the *P. koraiensis* were analyzed at different periods to reveal the impact of the PWN on the endophytes of pine trees. The study aimed to identify the dominant microorganisms at various periods and provide guidance and scientific basis for the development of biological control agents against PWD.

## 2 Materials and methods

### 2.1 Research location and sample selection

**Figure 1:**
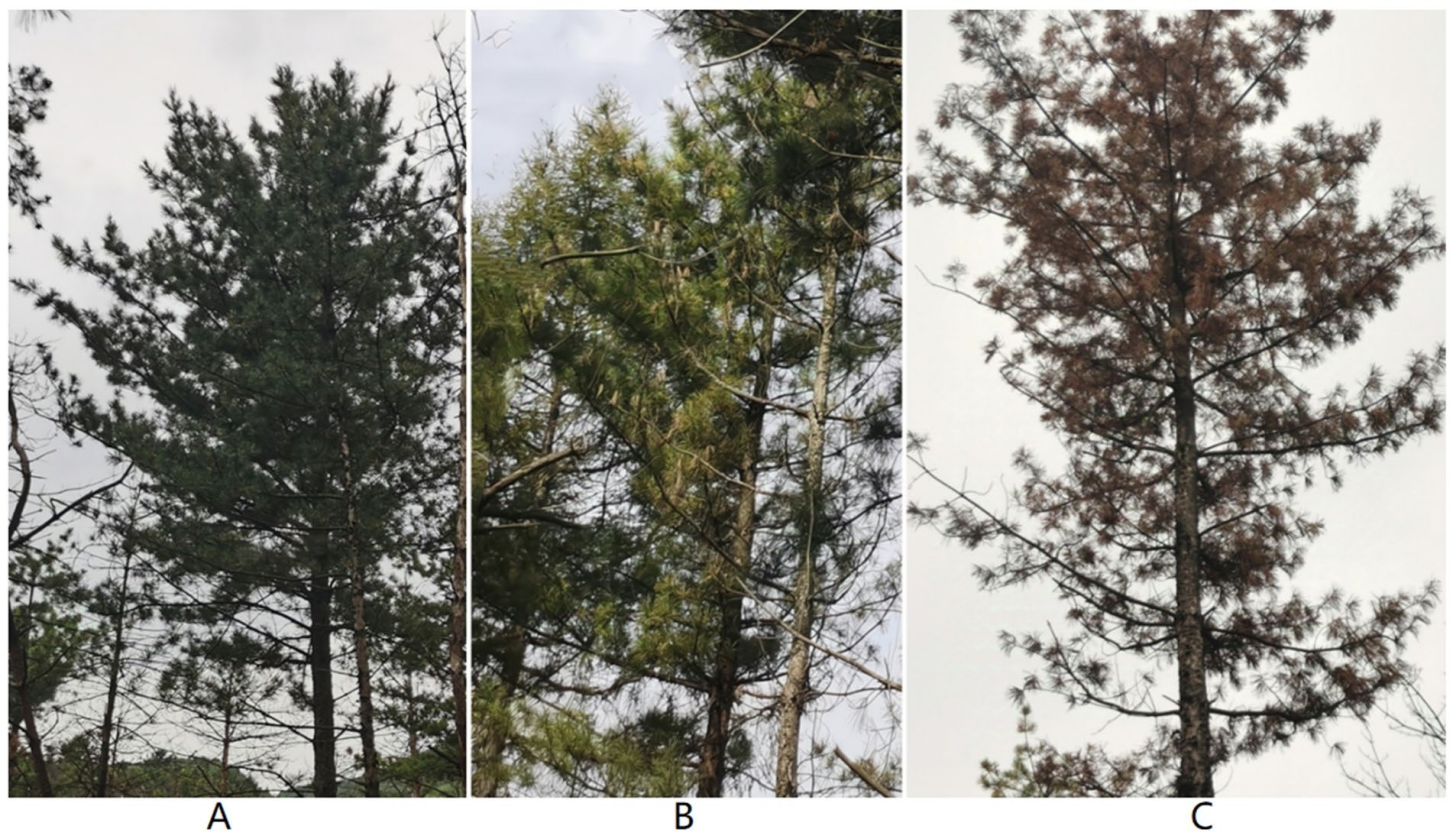
Healthy *P. koraiensis* (A), early-stage diseased *P. koraiensis* (B), and late-stage diseased *P. koraiensis* (C).

The sampling site is located at the Da Huo Fang Experimental Forest Farm in Fushun City, Liaoning Province, China (41°52′N, 123°55′E). The climatic characteristics are of a mid-temperate continental monsoon climate, with an average annual temperature of 6.6°C, an average annual precipitation of 750–850 mm, and an average frost-free period of 130–150 days. The soil type is neutral soil. Four *P. koraiensis* forests infected by the PWN were selected as sample plots. At each site, two healthy *P. koraiensis*, two *P. koraiensis* naturally infected by *B. xylophilus* at the early stage (PKe), and two *P. koraiensis* naturally infected by *B. xylophilus* at the last stage (PKl) were selected as samples, with the diseased trees being less than 15 meters away from the adjacent healthy trees (Figure 1). Healthy trees are defined as those with green needles throughout the entire plant and no presence of PWN within the tree body. Early-stage infection refers to a condition where the needles show slight wilting and browning, and the tree body contains PWN. Late-stage infection refers to a condition where the entire tree is dead, the needles are red, and the tree body contains PWN (Table 1).

**Table 1:** Comparison of symptom differences between PKh, PKe, and PKl.

Symptoms	PKh	PKe	PKl
Needle color	All needles are green	A few needles have turned yellow and wilted	All needles have turned yellow and red
Resin secretion	Normal	Reduced resin secretion	Resin secretion has ceased
Beetle infestation	No signs of beetle infestation on the tree	The tree shows signs of beetle infestation and egg-laying	The entire tree is densely covered with signs of beetle infestation, and beetle frass is visible

### 2.2 Sample collection

During September 2023, Samples were taken from 44-year-old *P. koraiensis* trees that have not been damaged by other diseases except for PWD. Sample collection was carried out in four different plots. In each plot, two healthy *P. koraiensis* (PKh), two *P. koraiensis* in the early stage of disease (PKe), and two *P. koraiensis* in the late stage of disease (PKl) were selected. A total of 24 trees were collected for sampling. Six parts from two trees of the same type, including needles, branches, upper trunk, middle trunk, lower trunk, and roots, were collected and mixed into one sample. In total, 12 samples were obtained for microbial community analysis, consisting of 3 period types per plot across 4 plots. All samples were placed in sterile bags, refrigerated, and then transported to the laboratory. The samples were subjected to surface disinfection treatment, then placed into 10 mL sterile centrifuge tubes and stored at −80°C. The processed samples were delivered to Shenyang Bosite Biotechnology Co., Ltd., where they were subjected to high-throughput sequencing methods using ITS and 16S DNA sequences to analyze the diversity of fungi and bacteria in the samples.

### 2.3 Extraction of total genomic DNA and PCR amplification

Genomic DNA was extracted using the CTAB method (Plomion et al., 1995). The DNA was then subjected to polymerase chain reaction (PCR) amplification of the V5-V7 region of the bacterial 16S rDNA gene using the primer pairs 799F (5′-AACMGGATTAGATACCCKG-3′) and 1193R (5′-ACGTCATCCCCACCTTCC-3′) and the ITSl region of the fungal ITS gene using primer pairs ITS1F (CTTGGTCATTTAGAGGAAGTAA) and ITS2R (GCTGCGTTCTTCATCGATGC) with the barcode. PCR was performed using TransStart Fastpfu DNA Polymerase on a TransGen Thermal Cycler (AP221-02, Beijing, China). PCR products were purified using the Agencourt AMPure XP nucleic acid purification kit and were used to construct a library and perform Miseq sequencing. The Uparse software was used to classify the sequences into many groups based on their similarity, with each group being an Operational Taxonomic Unit (OTU). OTUs were divided at different similarity levels, and bioinformatics statistical analysis was performed on OTUs at a 97% similarity level.

### 2.4 Species annotation

To obtain the taxonomic information corresponding to each OTU, methods such as the RDP Classifier algorithm (default), blast, or the uclust consensus taxonomy assigner were used to align and analyze the representative sequences of OTUs, and to annotate the community’s species information at various levels (kingdom, phylum, class, order, family, genus, species).

### 2.5 Data analysis

The obtained Fastq data were subjected to quality control processing to ultimately yield high-quality Fasta data. Clean tags were used to generate OTUs through clustering (or noise reduction) methods. To explore the trend of alpha diversity in samples with respect to sequencing depth, the software QIIME2 was used to plot rarefaction curves (Amato et al., 2013). By constructing Venn diagrams using statistical and plotting software in the R language, the number of OTUs in environmental samples and their overlaps between samples or groups can be visually represented (Fouts et al., 2012). The alpha diversity index values of the samples are calculated using QIIME scripts, and the main indicators of alpha diversity include Chao1 richness and Shannon’s index. Box plots at different levels can be drawn to clearly and directly understand the relative abundance of species between different treated samples (Schloss et al., 2011). Beta diversity indices focus on comparing the diversity between different habitats, that is, the differences between samples. By using the abundance information of species (ASV/OTU) in the samples and the evolutionary relationships between characteristic/representative sequences, multi-dimensional species data can be reduced to one-dimensional data—sample difference distances, thereby characterizing the community differences between these two samples from different perspectives. FUNGulid analysis, where FUNGulid = Fungi + Functional + Guild, is a database for functional annotation of fungi, currently covering over 12,000 fungal functional annotation records. The FAPROTAX script collapse_table.py is used to analyze bacterial species abundance.

## 3 Results

### 3.1 *Pinus koraiensis* endophytic fungi diversity analysis

#### 3.1.1 The number of endophytic fungal OTUs

**Figure 2:**
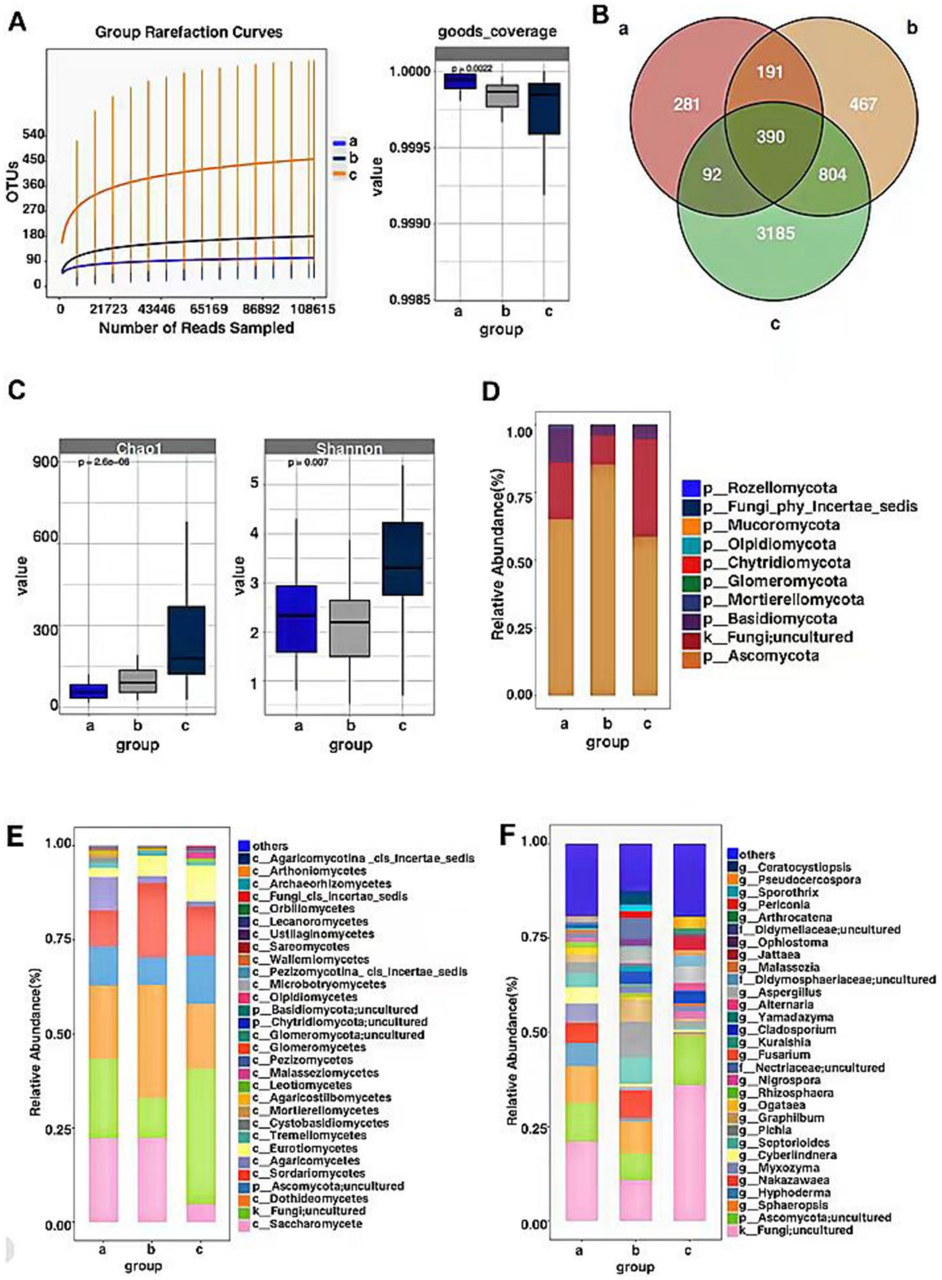
OTU-based rarefaction curve of endophytic fungal communities of *P. koraiensis* and coverage index (A), Venn diagram of OTU distribution of fungi in three stages of *P. koraiensi* (B), Abundance and diversity of endophytic fungi in *P. koraiensis* (C), Composition of endophytic fungal Phyla (D), Classes (E), Genera (F) in *P. koraiensis*.

Sequencing of the ITS gene in endophytic fungal communities of *P. koraiensis* samples across three periods resulted in a total of 15,457,470 effective sequences, with an average length of 269 base pairs for the optimized sequences. Based on the dilution curves and the fact that the library coverage was consistently above 99%, it indicates that the sample libraries in this study encompassed the majority of fungal taxa, reflecting the structural composition of the endophytic fungal communities. The results are representative of the true levels of endophytic fungi within the samples (Figure 2A). Groups a (PKl), b (PKe), and c (PKh) contain 954, 1852, and 4,471 OTUs, respectively. The unique OTUs are 281 in Group a, 467 in Group b, and 3,185 in Group c, accounting for 5.19, 8.63, and 58.87% of the total OTU count (5,410), respectively. The unique species abundance of PKh is the highest, accounting for more than half of the total number of species, while PKe and PKl have relatively fewer species, only about 5–8%. A total of 390 OTUs were shared across the three periods. The number of OTUs (804) common to PKh and PKe is significantly higher than the number of OTUs (92) common to PKh and PKl (Figure 2B). Analysis of the number of fungal OTUs associated with three states of *P. koraiensis* infected by the PWN reveals that the PWN has a significant impact on the endophytic fungi of *P. koraiensis*, causing a substantial decline in abundance after the onset of disease.

#### 3.1.2 Endophytic fungal alpha diversity

Through the analysis of the Chao1 index for the *P. koraiensis* endophytic fungal communities, the Chao1 index of sample a (PKl) was significantly lower than that of samples b (PKe) and c (PKh) (*p* < 0.05). Additionally, the Chao1 index of sample b was significantly lower than that of sample c (*p* < 0.05). Analysis of the Shannon index for the *P. koraiensis* endophytic fungal communities revealed that the Shannon index of sample c was significantly higher than that of samples a and b (*p* < 0.05). Additionally, the Shannon index of sample a was higher than that of sample b (*p* < 0.05) (Figure 2C). The diversity and richness of endophytic fungi in PKh are the highest, while PKe and PKl are not significantly different from each other. Analysis of both indices indicates that *B. xylophilus* has an impact on the alpha diversity and richness of endophytic fungi in *P. koraiensis*, with a trend of decreasing alpha richness as the extent of disease increases.

#### 3.1.3 Analysis of the composition of endophytic fungal communities

The OTU counts of the *P. koraiensis* endophytic fungal communities across three periods were classified by species, belonging to 10 phyla, 43 classes, and 519 genera. In samples from groups a (PKl), b (PKe), and c (PKh), the phylum Ascomycota was the most abundant, with abundances of 65.01, 85.26, and 58.57%, respectively. Next in abundance were the Fungi; uncultured phyla, with respective percentages of 21.03, 10.74, and 36.04%. Following these were the Basidiomycota, with abundances of 12.62, 3.84, and 4.94%. The phylum Mortierellomycota was found to be increased only in PKl, with a proportion higher than 1%, accounting for 1.04% (Figure 2D). It is evident that PWD has influenced the community composition of *P. koraiensis* endophytic fungi at the phylum level, and the number of Mortierellomycota has increased during the late stages of the disease. In all periods, the phylum Ascomycota had the highest content, exceeding more than 50% of the total abundance, and even surpassing 85% in the early stages of the disease. The abundance of Fungi; uncultured phyla also accounts for a certain proportion, approximately 10–36% of the total.

At the class level, in Group a, Saccharomycetes has the highest relative abundance at 22.23%, followed by Fungi; uncultured (21.03%), Sordariomycetes (9.69%), and Agaricomycetes (8.77%). In Group b, the relative abundance of Dothideomycetes is the highest, at 29.97%, followed by Saccharomycetes (22.31%), Sordariomycetes (19.78%), Fungi; uncultured (10.74%), and Ascomycota; uncultured (7.27%). In Group c, the relative abundance of Fungi; uncultured is the highest, at 36.04%, followed by Dothideomycetes (17.18%), Sordariomycetes (12.91%), Ascomycota; uncultured (12.90%), and Eurotiomycetes (9.52%). Tremellomycetes are present in more than 1% only in diseased *P. koraiensis*, while Cystobasidiomycetes and Mortierellomycetes are present in more than 1% only in PKl (Figure 2E). The PWN can influence the community composition ratios at the fungal class level, and when *P. koraiensis* is healthy, the abundance of Fungi; uncultured is the highest. In the early stages of disease, the class Dothideomycetes has the highest richness, and there is an increase in the abundance of the Taphrinomycetes class compared to healthy *P. koraiensis*. In the late stages of disease, the Saccharomycetes has the highest abundance, and there is an increase in the abundance of both the Cystobasidiomycetes, Basidiomycetes and Mortierellomycetes.

At the genus level, the genera with relatively higher abundance in Group a include *Fungi; uncultured* (21.03%), *Ascomycota; uncultured* (10.34%), *Sphaeropsis* (9.68%), *Hyphoderma* (6.05%), *Nakazawaea* (5.38%), *Myxozyma* (5.10%), *Cyberlindnera* (4.44%), *Septorioides* (3.73%), *Pichia* (2.88%), *Graphilbum* (2.08%), *Rhizosphaera* (1.58%), Nigrospora (1.16%), Nectriaceae; uncultured (1.04%). In Group b, the genera with relatively higher abundance include *Fungi; uncultured* (36.04%), *Pichia* (9.41%), *Sphaeropsis* (8.40%), *Nakazawaea* (7.41%), *Ascomycota; uncultured* (7.27%), *Septorioides* (7.09%), *Didymellaceae; uncultured* (5.37%), *Aspergillus* (3.94%), *Ceratocystiopsis* (3.53%), *Cladosporium* (3.29%), *Ophiostoma* (1.90%), *Periconia* (1.79%), *Sporothrix* (1.76%), *Yamadazyma* (1.65%), *Nectriaceae; uncultured* (1.31%), *Selenophoma* (1.14%), *Pestalotiopsis* (1.07%), *Rhizosphaera* (1.05%). In Group c, the genera with relatively higher abundance include Ascomycota; uncultured (12.90%), *Fungi; uncultured* (10.74%), *Aspergillus* (4.49%), *Jattaea* (3.59%), *Cladosporium* (3.23%), *Pseudocercospora* (3.07%), *Didymosphaeriaceae; uncultured* (2.76%), *Phaeococcomyces* (2.05%), *Nigrospora* (1.99%), *Pichia* (1.78%), *Malassezia* (1.51%), *Coryneliaceae_gen_Incertae_sedis* (1.37%), *Arthrocatena* (1.32%), *Nectriaceae; uncultured* (1.27%), *Candida* (1.02%) (Figure 2F). PWD has affected the compositional ratios of *P. koraiensis* endophytic fungi at the genus level, with *Fungi; uncultured* consistently having the highest abundance across all periods. Fungal genera such as *Fungi; uncultured*, *Graphilbum*, *Pichia*, *Aspergillus* and *Cladosporium* are more significantly impacted by the infection of the PWN.

#### 3.1.4 Endophytic fungal beta diversity

**Figure 3:**
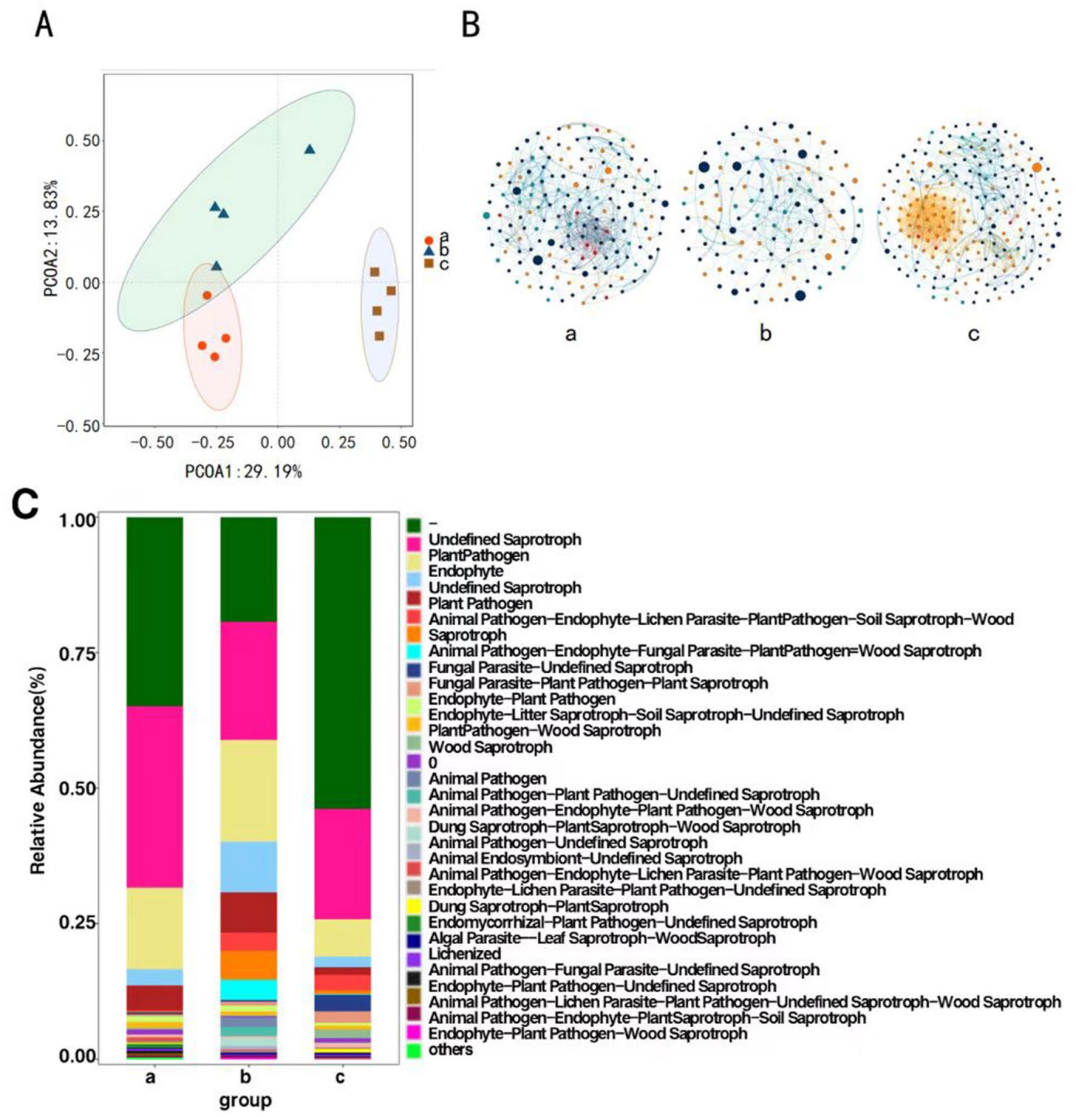
Principal coordinate analysis of endophytic fungi of *P. koraiensis* (A), The MENs of endophytic fungal community in *P. koraiensis* (B), Bar graph of functional abundance of endophytic fungi of *P. koraiensis* (C).

Principal Coordinates Analysis was conducted on the composition of *P. koraiensis* endophytic fungal communities across three periods using the Bray-Curtis method to assess the differences in community composition. The contribution rates of the first principal coordinate (PCoA1) and the second principal coordinate (PCoA2) are 29.19 and 13.83%, respectively, (*p* < 0.05), with a combined contribution rate of 43.02%. At the OTU level, samples from Group a and Group b are closer to each other, indicating a higher similarity in community composition. The four samples from Group c are distinctly clustered and are further apart from the samples from Groups a and b, suggesting that *B. xylophilus* may alter the original community structure to varying degrees in diseased *P. koraiensis*. There are significant differences in the endophytic fungal communities among Groups a, b, and c (Figure 3A).

#### 3.1.5 Correlation of dominant species

Network structure analysis was conducted on the endophytic fungi of *P. koraiensis* across three different periods, and a visual diagram of the fungal molecular ecological network was constructed. The co-occurrence network of endophytic fungal communities underwent significant changes under the *P. koraiensis* environment during different periods. Firstly, the complexity of the network decreases due to infection with PWD, with the complexity being the lowest in the early stages of the disease. In the endophytic microbial networks for Group a (PKl), Group b (PKe), and Group c (PKh), the number of connected nodes are 164, 116, and 218 respectively, and the number of interactions, or edges, are 586, 254, and 1,022, respectively. There is a reduction in the number of nodes and the connections, or edges, within the co-occurrence networks of the endophytic microbes. In the overall connections, both b and c show positive correlation relationships, while in a, only *Sphaeropsissapinea* and *Hyphodermasetigerum* exhibit a negative correlation relationship, with the rest showing positive correlations (Figure 3B). The relationships among endophytic fungi in *P. koraiensis* across the three periods are primarily cooperative and synergistic, rather than competitive.

#### 3.1.6 Functional prediction of fungal communities

Functional prediction analysis of endophytic fungi was conducted using FUNGuild. The main functions predicted in the samples include Undefined (−), Undefined Saprotroph, plant pathogen, Animal Endosymbiont-Undefined Saprotroph and Endophyte. In Group a (PKl) and Group c (PKh), the abundance of the Undefined function (−) is the highest. In Group b (PKe), the abundance of Undefined Saprotroph is the highest (Figure 3C).

### 3.2 *Pinus koraiensis* endophytic bacteria diversity analysis

#### 3.2.1 Sample endophytic bacterial OTU count

**Figure 4:**
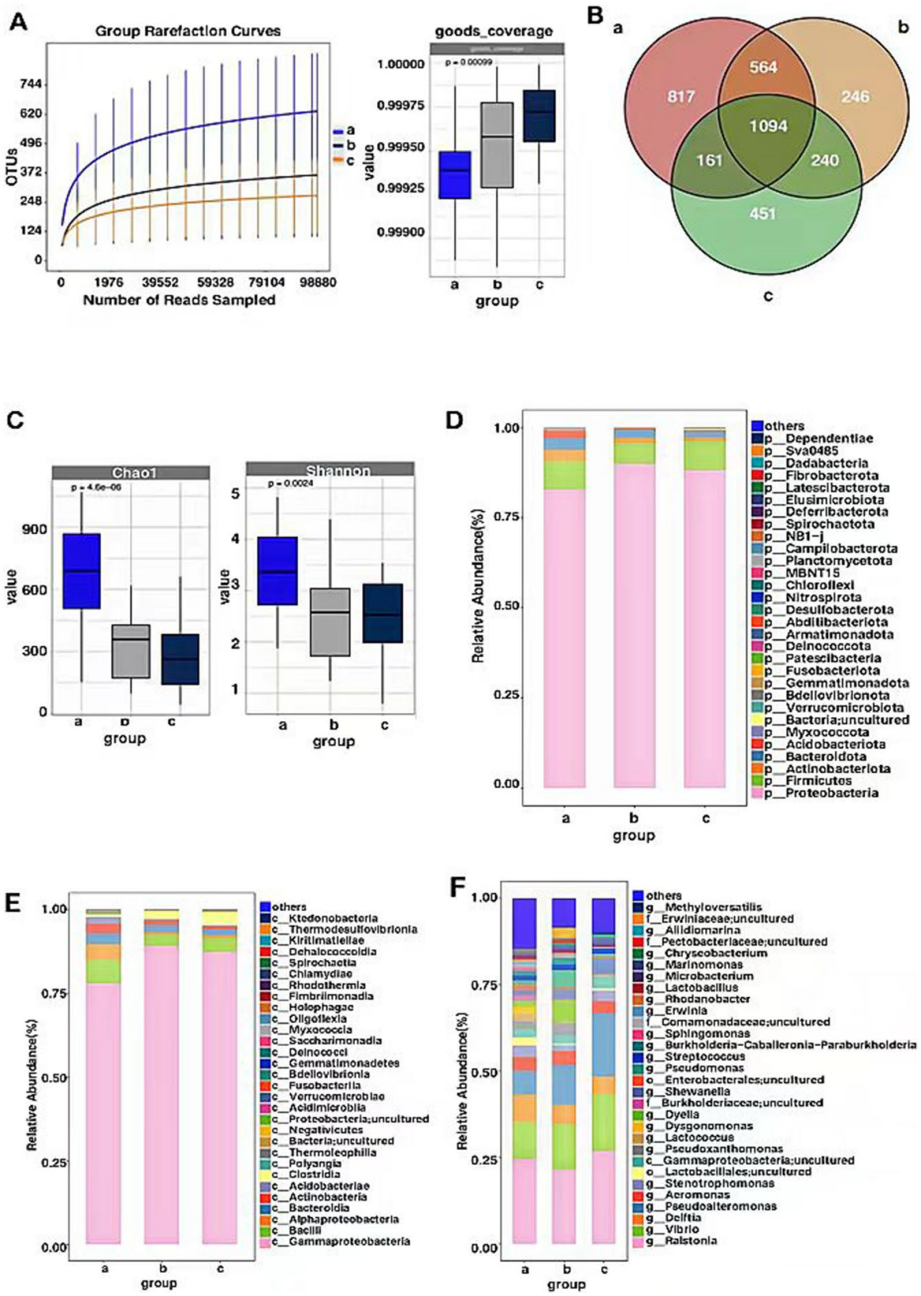
OTU-based rarefaction curve of endophytic bacterial communities of *P. koraiensis* and coverage index (A), Venn diagram of OTU distribution of bacteria in three stages of *P. koraiensi* (B), Abundance and diversity of endophytic bacteria in *P. koraiensis* (C), Composition of endophytic bacterial Phyla (D), Classes (E), Genera (F) in *P. koraiensis*.

Sequencing of the 16SrDNA gene in endophytic bacterial communities of *P. koraiensis* samples across three periods resulted in a total of 13,756,325 effective sequences, with an average length of 301 base pairs for the optimized sequences. Based on the dilution curves and the fact that the library coverage was consistently above 99%, it indicates that the sample libraries in this study encompassed the majority of bacterial taxa, reflecting the structural composition of the endophytic bacterial communities. The results are representative of the true levels of endophytic bacteria within the samples (Figure 4A). Groups a (PKl), b (PKe), and c (PKh) contain 2,639, 2,144, and 1,946 OTUs, respectively. The unique OTUs are 817 in Group a, 246 in Group b, and 451 in Group c, accounting for 5.19, 8.63, and 58.87% of the total OTU count (3576), respectively. The shared OTUs are 1,094, accounting for 30.6% of the total (Figure 4B). The analysis of the number and changes of bacterial OTUs in the three states indicates that *B. xylophilus* affects the abundance of endophytic bacteria in *P. koraiensis*, and infection leads to an increase in abundance.

#### 3.2.2 Endophytic bacterial alpha diversity

Through the analysis of the Chao1 index for the *P. koraiensis* endophytic bacterial communities, it was found that the Chao1 index of sample a (PKl) was significantly higher than that of samples b (PKe) and c (PKh) (*p* < 0.05), and the Chao1 index of sample b was higher than that of sample c (*p* < 0.05). Analysis of the Shannon index for the *P. koraiensis* endophytic bacterial communities revealed that the Shannon index of sample a was significantly higher than that of samples b and c (*p* < 0.05), and the sample b was lower than that of sample c (*p* < 0.05) (Figure 4C). The diversity and abundance of endogenous bacteria in PK1 are the highest, while PKe and PKh are not significantly different from each other. Two types of index analysis both indicate that *B. xylophilus* has an impact on the alpha diversity and abundance of endogenous bacteria in *P. koraiensis*, and as the degree of infection increases, the alpha diversity shows an increasing trend.

#### 3.2.3 Analysis of the composition of endophytic bacterial communities

The OTU counts of the *P. koraiensis* endophytic bacterial communities across three periods were classified by species, belonging to 33 phyla, 90 classes, and 758 genera. In samples from groups a (PKl), b (PKe), and c (PKh), the phylum Proteobacteria was the most abundant, with abundances of 82.75, 89.95, and 88.25%, respectively. Next in abundance were the Firmicutes phyla, with respective percentages of 7.90, 5.68, and 8.00%. Following these were the Bacteroidota, with abundances of 3.21, 2.16, and 1.75%. In groups a and b, the proportion of the phylum Actinobacteriota is higher than 1%, being 3.19 and 1.52%, respectively. The phylum Acidobacteriota is only in group a where its proportion exceeds 1%, specifically 2.03% (Figure 4D). It is known that *B. xylophilus* affects the composition ratio of endophytic bacterial communities at the phylum level in *P. koraiensis*, and when in the late stages of the disease, the abundance of Actinobacteriota and Acidobacteriota increases. In all periods, the phylum Proteobacteria is *P. koraiensis* the most abundant, accounting for more than 50% of the total abundance, and even exceeding 85% in the early stages of infection. The phylum Firmicutes has a relatively stable abundance, accounting for about 5–8% of the total abundance.

At the class level, in Group a, Gammaproteobacteriahas the highest relative abundance at 77.95%, followed by Bacilli (6.91%), Alphaproteobacteria (4.83%), Bacteroidia (3.16%), actinobacteria (2.93%), Acidobacteriae (1.98%). In Group b, the relative abundance of Gammaproteobacteria is the highest, at 87.72%, followed by Bacilli (3.45%), Clostridia (2.72%), Bacteroidia (2.20%), Actinobacteria (1.73%), Alphaproteobacteria (1.24%). In Group c, the relative abundance of Gammaproteobacteri is the highest, at 86.97%, Bacilli (4.16%), Clostridia (3.53%), Bacteroidia (1.73%), actinobacteria (1.18%) (Figure 4E). The PWD significantly affects the community composition ratio at the bacterial class level, with Gammaproteobacteria having the highest abundance, approximately 80% in all three periods, and Bacilli also have a relatively rich abundance, but only about 4–7%. In the late stages of the disease, the abundance of the Acidobacteria phylum has increased, reaching 1.98%.

At the genus level, the genera with relatively higher abundance in Group a include *Ralstonia* (24.19%), *Vibrio* (10.88%), *Delftia* (7.76%), *Pseudoalteromonas* (6.93%), Stenotrophomonas (3.34%), *Lactobacillales; uncultured* (2.39%), *Gammapr-oteobacteria; uncultured* (2.34%), *Pseudoxanthomonas* (2.21%), *Lactococcus* (2.15%), *Dysgonomonas* (2.03%), *Pseudomonas* (1.71%), *Dyella* (1.67%), *Burkholderiaceae; uncultured* (1.59%), *Shewanella* (1.55%), *Enterobacterales; uncultured* (1.47%), *Strep-tococcus* (1.36%), Sphingomonas (1.08%). In Group b, the genera with relatively higher abundance include *Ralstonia* (19.99%), *Vibrio* (14.19%), *Pseudoalteromon-as* (11.61%), *Dyella* (5.76%), *Delftia* (5.02%), *Pseudomonas* (4.70%), *Pseudoxantho-monas* (3.26%), *Shewanella* (2.72%), *Erwiniaceae; uncultured* (2.69%), *Gammaprote-obacteria; uncultured* (2.52%), *Streptococcus* (1.77%), *Stenotrophomonas* (1.70%), *Pect-obacteriaceae; uncultured* (1.10%). In Group c, the genera with relatively hig-her abundance include *Ralstonia* (24.81%), *Pseudoalteromonas* (17.85%), *Vibrio* (17.81%), *Delftia* (5.11%), *Shewanella* (4.13%), Aeromonas (4.11%), *Gammaproteoba-cteria; uncultured* (3.37%), *Stenotrophomonas* (2.93%), *Streptococcus* (1.71%), *Mari-nomonas* (1.52%), *Pseudomonas* (1.08%), *Methyloversatilis* (1.00%) (Figure 4F). Afte-r *P. koraiensis* was infected by *B. xylophilus*, the disease affected the composi-tional ratios of endophytic bacteria within the pines. Across all periods, *Ralsto-nia* was consistently the most abundant, accounting for about 20–25% of the c-ommunity. Other bacterial genera such as *Pseudoalteromonas*, *Shewanella*, and *Pseudomonas* were also significantly affected by the *B. xylophilus* infection.

#### 3.2.4 Endophytic bacterial beta diversity

**Figure 5:**
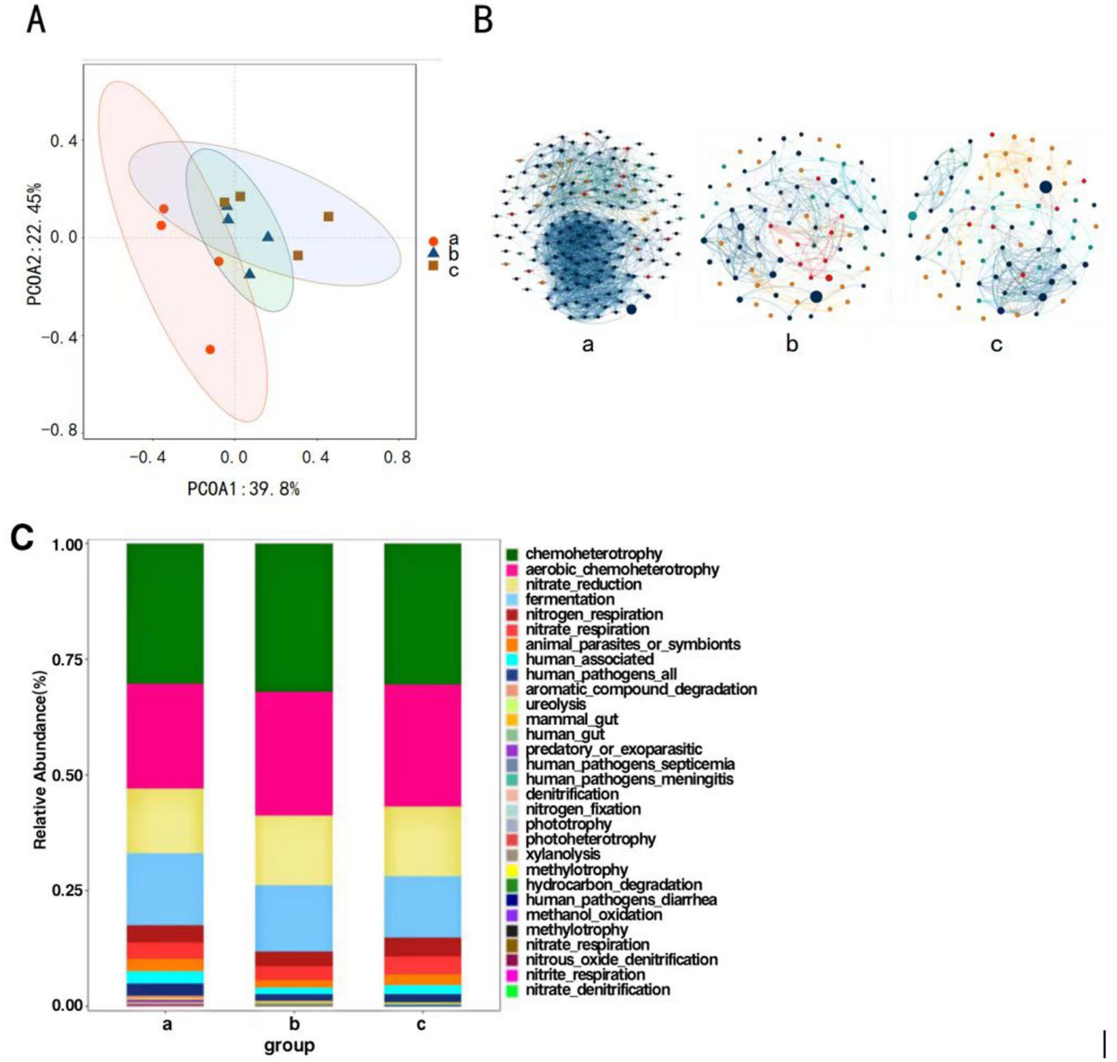
Principal coordinate analysis of endophytic bacteria of *P. koraiensis* (A), The MENs of endophytic bacterial community in *P. koraiensis* (B), Bar graph of functional abundance of endophytic bacteria of *P. koraiensis* (C).

Principal Coordinates Analysis was conducted on the composition of *P. koraiensis* endophytic bacterial communities across three periods using the Bray-Curtis method to assess the differences in community composition. The contribution rates of the first principal coordinate (PCoA1) and the second principal coordinate (PCoA2) are 39.8 and 22.45%, respectively, (*p* < 0.05), with a combined contribution rate of 62.25%. The four samples from groups a, b, and c are all relatively concentrated, with groups b and c being closer to each other. This indicates that the pine wood nematode can alter the community structure of red pines, and there are significant differences in the composition of endophytic bacterial communities among groups a, b, and c (Figure 5A).

#### 3.2.5 Correlation of dominant species

Network structure analysis was conducted on the endophytic bacteria of *P. koraiensis* across three different periods, and a visual diagram of the bacterial molecular ecological network was constructed. The co-occurrence network of endophytic bacterial communities underwent significant changes under the *P. koraiensis* environment during different periods. First, the complexity of the network increases with the severity of the disease. In the endophytic microbial networks for Group a (PKl), Group b (PKe), and Group c (PKh), the number of connected nodes are 166, 87, and 81 respectively, and the number of interactions, or edges, are 1925, 342, and 326, respectively. As the severity of the disease intensifies, both the number of nodes and the connections, or edges, within the co-occurrence networks of the endophytic microbes increase. In the total connections, both a and b show positive correlation relationships, while in c there are 36 pairs of negative correlations, but the predominant relationship is positive (Figure 5B). The relationships among endophytic bacteria in *P. koraiensis* across the three periods are primarily cooperative and synergistic, rather than mutually exclusive.

#### 3.2.6 Functional prediction of bacterial communities

Functional prediction analysis of endophytic bacteria was conducted using FAPROTAX. The main functions predicted in the samples include chemoheterotrophy, Aerobic Chemoheterotrophy, fermentation, nitrate _ reduction and nitrogen respiration (Figure 5C). In all periods, the chemoheterotrophic function is the most abundant, and the overall functional composition of the three periods is essentially similar, with the community structures being relatively close to each other.

## 4 Discussion

In this study, the determination of endophytes in *P. koraiensis* of different disease grades confirmed the diversity and richness of endophytic microorganisms in *P. koraiensis*. In the classification of fungal OTUs, PKh has the highest number of unique OTUs. Studies have shown that the fungal diversity in healthy *Pinus massoniana* trees is higher than that in dead *P. massoniana* (Yin et al., 2021), which is consistent with this article. Yin et al. (2021) studied the structural changes of endophytic bacterial flora in *P. massoniana* seedlings after infection by *B. xylophilus*, and found that the structural changes of endophytic bacterial flora in the stems of *P. massoniana* were extremely significant. In the study of community structure, endophytic fungi are dominated by Ascomycota, both Ascomycota and Basidiomycota are common endophytic fungi in plants (Huang, 2017), and Ascomycota and Basidiomycota are the main dominant groups in the endophytic fungal community of pine trees (Deng et al., 2023; Zheng, 2022). The phylum Ascomycota is the most important group among pine tree fungi, which is the same as the results of other plant studies (Li et al., 2020; Xie, 2021; Xie et al., 2020; Yu et al., 2023; KembelSteven and MuellerRebecca, 2014). Among the isolated and purified strains, the genera with separation frequency greater than 5% were dominant genera, and *Trichoderma*, *Penicllium*, *Mucor*, and *Mortierella* were dominant genera, most of which were saprophytic bacteria. This is consistent with previous conclusions of pure culture studies on endophytic fungi in pine trees Eo et al., 2018. In the classification of bacterial OTUs, PKl has the highest number of unique OTUs. Endophytic bacteria are dominated by the phylum Proteobacteria, which is consistent with the results of the study on *P. massoniana* endophytic microorganisms (Yuan et al., 2021). There are differences in the richness of microbial communities, community structure composition, and functional diversity between the rhizosphere microorganisms and root endophytes of *Pinus dabeshanensis* (Xiang et al., 2024). In other studies of Pinus genus endophytic bacteria, the dominant strains of endophytic bacteria obtained through isolation and purification are consistent with the conclusions of this study (Hu et al., 2019). The decline in endophytic microbial diversity and abundance leads to an increase in disease infection, which is consistent with the conclusions drawn from the study of phytoplasma on the host’s root endophytic bacteria (Qiu et al., 2019). It was found that the invasion of *B. xylophilus* significantly changed the endophytic microbial structure of *P. koraiensis*. With the passage of infection time, the diversity of bacteria showed a decreasing trend of fungi and an increasing trend of bacteria. The results of the endophytic microbial community structure composition analysis indicate that after being invaded by *B. xylophilus*, there will be significant changes in the composition of the dominant endophytic bacteria in *P. koraiensis*. The PCOA analysis results at the OTU level further demonstrate that the structure of the endophytic microbial community in *P. koraiensis* has undergone significant changes after invasion by *B. xylophilus*.

In plant tissues, the interactions between microorganisms are diverse and dynamic, exerting various effects and influences on the structure and function of microbial communities (Karoline and Jeroen, 2012). Through mutual exchange of materials, signal transduction, energy flow, and other factors, they form a complex and stable dynamic ecological network. The construction of microbial molecular ecological network analysis is of great significance for further understanding and exploring the structure of microbial communities (Deng et al., 2012). In different parts, the proportion of positive correlations in the microbial ecological network structure is much higher than that of negative correlations (Krings et al., 2007), and the fungi exhibit a synergistic development relationship within plants (Yan et al., 2015). The network structure of the late-stage diseased *P. koraiensis* is more complex, and the bacterial ecology after disease is more susceptible to external disturbances. The proportion of positive correlations in the network structure is much higher than that of negative correlations, which is consistent with the synergistic symbiotic relationship of bacteria within plants (Peix et al., 2015). Some endophytic bacteria isolated from maritime pine exhibit strong antagonistic activity with endophytic fungi and bacteria, indicating that there are also mutual exclusion relationships among endophytes.

Endophytic microorganisms within plants are a natural part of the plant’s micro-ecosystem and can coexist with the plant body for a long time. They have characteristics such as rapidity, sensitivity, and specificity in response to pests and diseases. In general, endophyte-plant interactions can be influenced by both biotic and abiotic factors, including the host plant’s locale and genotypes, environmental conditions, and biological species that interact with the host plant (Zhao et al., 2024). Endophytic microorganisms are widely present in pines and can respond to external environmental stimuli such as PWD (Li, 2018). Apart from PWD, other communities that may affect the interactions between endophytes and pines include the rhizosphere microbiome, pathogenic microbes of the host plant, and plant-feeding insects (Zhao et al., 2024). Many studies have shown that after plants are infected by pathogens, it affects the host’s microbial community (Douanla-Mel et al., 2013), their resistance to diseases decreases, and a large number of external bacterial communities are more likely to invade the plants, leading to an increase in the types of endophytic bacteria, which may be the reason for the increase in the abundance of other bacterial genera. The increase in endophytic bacterial diversity may be due to the disease causing the plant to lose its ability to resist microbial invasion or to lose control over the growth of endophytic microorganisms (Liu et al., 2018). Studies have shown that Proteobacteria are common plant pathogens and parasites in plant tissues and can cause a variety of plant diseases. In addition, experiments have also found that the abundance of Firmicutes also increases, but not significantly, and some literature has pointed out that anaerobic environments are the preferred growth environment for Firmicutes (Mandal and Jeon, 2023), which may be related to the changes in the host’s internal environment caused by the invasion of *B. xylophilus*. Some endophytic microorganisms may have potential biocontrol effects on pine wilt disease. Tan et al. (2003) obtained a strain of *Bacillus firmus* GD2 through experiments, which can affect the infection of pine wilt disease, and proved for the first time that there is a close relationship between pine endophytic bacteria and pine wilt disease. Zhao et al. (2000) proposed that both bacteria and pine wood nematode are indispensable pathogenic factors of pine wood nematode disease, and control pine wood nematode disease by antibiotics to control pathogenic bacteria. Li et al. (2016) isolated a strain of antagonistic bacteria *Bacillus pumilus* against *B. xylophilus* from the stems of *P. massoniana*, which provided a theoretical basis for the practical application of the bacteria in the future. *Serratia marcescens* isolated from the pupal chamber of *P. massoniana* can promote the death of *M. alternatus*, and *Serratia* is often isolated from *P. massoniana* (Zhang et al., 2021). Song et al. (2020) isolated a strain of *Pantoea eucalypti* from pine trees, and speculated that the strain could promote the growth of pine trees according to its genomic characteristics and carrying function. Exploring the structural changes of endophytic flora of *P. koraiensis* after natural infection with PWN is helpful to provide theoretical reference for the early diagnosis of pine wood nematode disease and the excavation of biocontrol strains in the later stage.

This study collected endophytic microorganisms from *P. koraiensis* by using the method of sampling large trees of naturally grown *P. koraiensis* outdoors. For the diversity study of endophytes in healthy *P. koraiensis* and *P. koraiensis* in the early and late stages of PWD infection, conventional sequencing methods combined with high-throughput sequencing can achieve a good detection rate of species. This study only sampled and tested large trees of naturally grown healthy and naturally diseased *P. koraiensis*, obtaining the species diversity of endophytic microorganisms in mature *P. koraiensis*. In the future, further research can be conducted by artificially inoculating *B. xylophilus* into *P. koraiensis* seedlings, which can more accurately grasp the impact of PWD on the endophytic microorganisms of *P. koraiensis*.

## 5 Conclusion

In summary, the PWN significantly affected the abundance of endophytic microbes in *P. koraiensis*. As the severity of the disease increased, a significant downward trend was observed in the abundance of endophytic fungi, while an upward trend was noted in the abundance of endophytic bacteria. PWD significantly altered the composition of endophytic fungi in *P. koraiensis*, with Ascomycota being the most abundant phylum of endophytic fungi, and Proteobacteria being the most abundant phylum of endophytic bacteria. Since the endophytic resources of *P. koraiensis* remain largely unexplored, future research may focus on identifying potential endophytes that can serve as biocontrol agents against PWN.

## Data Availability

The original contributions presented in the study are included in the article/supplementary material, further inquiries can be directed to the corresponding author.
